# Transactions of the Ohio State Dental Society

**Published:** 1883-05

**Authors:** 


### Editorial.

### Transactions of the Ohio State Dental Society.

The volume of transactions of this society for the meeting of 1882, held in Columbus, December last, has just been issued.

It is a well gotten up volume, of 142 pages, and presents a full and clear statement of the doings of that body.

It contains much valuable matter, and should be at least in the hands of every dentist in the State.

It can be obtained of Ransom & Randolph, Toledo; Cogswell & Geer, Cleveland; Geo. W. Fels, and Spencer & Crocker, Cincinnati, Ohio, and J. H. Warner, Secretary, Columbus, Ohio. Send five cents for postage.

All such works are valuable and every progressive member of the profession will seek to have them.

